# Nonunion of subtrochanteric fractures: Comminution or Malreduction

**DOI:** 10.12669/pjms.323.9897

**Published:** 2016

**Authors:** Sang Hyun Park, Gyu Min Kong, Byeong Ho HA, Jun Ho Park, Kun Hyung Kim

**Affiliations:** 1 Sang Hyun Park, MD. Department of Urology, Haeundae Paik Hospital, Inje University, Busan, Republic of Korea; 2 Gyu Min Kong, MD. PhD. Department of Orthopaedic Surgery, Busan Paik Hospital, Inje University, Busan, Republic of Korea; 3 Byeong Ho HA, MD. Department of Orthopaedic Surgery, Busan Paik Hospital, Inje University, Busan, Republic of Korea; 4 Jun Ho Park, MD. Department of Orthopaedic Surgery, Busan Paik Hospital, Inje University, Busan, Republic of Korea; 5 Kun Hyung Kim, MD. PhD. Department of Occupational and Environmental Medicine and Institute of Environmental and Occupational Medicine, Busan Paik Hospital, Inje University, Busan, Republic of Korea

**Keywords:** Intramedullary nail, Nonunion, Subtrochanteric femur fracture

## Abstract

**Objective:**

This study aimed to investigate the impact on nonunion of the extent of comminution and postoperative displacement in patients surgically treated for subtrochanteric fractures.

**Methods:**

From 2008 to 2013, 44 patients with subtrochanteric fractures underwent surgery and follow-up. Retrospective data collection showed that it had 32 male and 12 female. Their mean age was 45 years. The case distribution according to Seinsheimer classification was as follows: IIA,8; IIB, 5; IIC, 7; IIIA, 8; IIIB, 3; IV, 9; and V, 4. Cephalomedullary nails were used in 28 cases; ordinary nails, in 9; and plates, in 7. After surgery, the fractures were evaluated for displacement on anteroposterior (AP) and lateral radiography.

**Results:**

Of the 44 patients, 37 achieved union from primary surgery at a mean time of 8.4 months. Five cases did not show union within the follow-up period. Two cases of nail breakage were diagnosed as non-union. Among the non-union cases, two were Seinsheimer classification IIIA; 3, IV; and 2, V. Displacement was observed on the lateral and A Pradiographs of 4 cases, on only the lateral radiographs of two cases, and in neither radiograph of one case. The risk of non-union was approximately 15.4 and 24.2 times higher when displacement was observed on the AP (95% confidence interval [CI]: 1.33–176.82) and lateral images (95% CI: 1.76–335.67), respectively.

**Conclusion:**

When displacement occurred after surgical treatment for subtrochanteric fractures, the risk of nonunion increased owing to the difficulty achieving stable fixation.

## INTRODUCTION

Anatomically, surgical reduction is difficult in subtrochanteric femur fractures because of the action of muscles that generate various deforming forces. Biomechanically, considerable stress is applied to these fractures during body-weight loading, and because they also involve cortical bone, which has poor ability to achieve union, the time to union is relatively long, with a high risk of nonunion or implant failure.^1^-^4^ Subtrochanteric fractures show two age-dependent peaks in frequency, with fractures in younger patients caused by high-energy traumas, such as road traffic accidents or falling from a high elevation, and fractures in elderly patients caused by minor falls accompanied by osteoporosis.^5^,^6^

Factors that increase the risk of nonunion in femoral fractures include age, comorbid medical diseases, smoking, extent of comminution, osteoporosis, quality of reduction, and fixation stability. Of these factors, only quality of reduction and fixation stability can be controlled by the surgeon.^7^-^11^ The aim of treatment of subtrochanteric fractures is to achieve union by preserving vascularity to the fracture while ensuring internal fixation. The surgical method most commonly considered to achieve this is intramedullary (IM) nailing.^12^,^13^ However, the medullary canal of the subtrochanteric area in the femur is broad and the proximal fragment is relatively short, which means that malreduction can occur easily. Malreduction needs to be taken into account during surgery, as it can affect treatment outcomes.^4^,^14^

The present study aimed to confirm the hypothesis that the possibility of nonunion is higher in more severely comminuted fractures and nonunion is more common in fractures with greater displacement after surgery.

## METHODS

In this study retrospective data was collected. It had 44 patients who underwent surgery for a subtrochanteric fracture between January 2008 and December 2013, and were available for at least one year of follow-up. Pathologic fractures, periprosthetic fractures and atypical fractures were excluded. Of the patients, 32 were male and 12 were female. Their mean age was 45 years (range, 18–70 years). Of the 44 cases, 23 were incurred from car accidents; 12, from falling from high elevation; and 9, from falling while walking.

The case distribution according to Seinsheimer classifications was as follows: II,20; IIIA, 8; IIIB, 3; IV, 9; V,4. Cephalomedullary nails were used in 28 cases; ordinary nails, in 9; and plates, in 7 cases. Postoperative displacement of the fracture was examined on AP and lateral radiographic images. Radiological examinations were then performed every month to check for union. By using the markings provided on the picture archiving and communication system (PACS) console (Maroview; Marotech, Seoul, South Korea), movement of the cortical bone by at least 5mm from the location of the fracture was defined as displacement. Union was defined as the formation of at least three cortical bridges on AP and lateral images.

Nonunion was defined as implant breakage, fixation failure (due to bone resorption, etc.), or lack of union within 6 months after surgery. Radiographic images were observed by two of the authors, who then reached a consensus regarding the interpretation.

The relationships between nonunion, patients’ general characteristics (sex, alcohol intake, and cigarette smoking), and fracture-related characteristics (traumatic history and Seinsheimer classification, AP and lateral displacements, and operation method) were evaluated by using the chi-square test and Fisher exact test. Next, multiple logistic regression analysis was used to evaluate the association between the factors that were found to be significant in the univariate analysis. Age was also entered in the multiple logistic regression analyses as a predictive variable. A best-fit regression model was built by using forward stepwise selection. All statistical analyses were performed with IBM SPSS Statistics version 21.0 (IBM Corp., Armonk, NY, USA). The statistical significance level was set at p<0.05.

## RESULTS

Of the 44 patients, 37 achieved union after the primary surgery, at a mean time to union of 8.4 months. Five patients did not achieve union within the follow-up period, and two patients experienced nail breakage. In these cases, the mean time from primary to secondary surgery was 8 months.

Five of the 7 patients who had nonunion had damage only to the femur. One case was accompanied by pelvic fracture, and another case was accompanied by fracture of the ipsilateral tibia. In terms of Seinsheimer classification, 2 cases were IIIA, 3 cases were IV, and two cases were V. Displacement was observed on both the AP and lateral radiographic images of 4 patients, on only the lateral radiographic images of 2 patients, and on neither radiographic image of one patient (Table-I).

**Table-I:** Data of non-union cases.

*Case*	*Sex/Age*	*Type of Fracture*	*Displacement Coronal/sagittal*	*Primary implant*	*Cause of injury*	*Combined injury*	*Smoking*	*Treatment for nonunion*
1	M/60	III A	-/+	CMN	TA	-	-	Additional plate/ AIBG
2	M/58	V	+/+	CMN	TA	-	+	Change to plate/AIBG
3	M/55	III A	+/+	CMN	PTA	-	+	Change to plate/AIBG
4	M/55	IV	+/+	CMN	TA	-	+	Additional plate/AIBG
5	M/32	IV	+/+	ON	MTA	-	-	AIBG
6	M/18	IV	-/+	ON	MTA	Pelvic fx	+	IM Nail change/AIBG
7	M/62	V	-/-	CMN	Fall	Tibia fx	-	Additional plate/AIBG

CMN: Cephalomedullary nail, ON: ordinary nail, TA: Traffic accident (car collision),

PTA: pedestrian traffic accident, MTA: motorcycle accident, AIBG: autogenous iliac bone graft.

Comorbid medical diseases did not affect nonunion, nor did sex, smoking, or drinking. Compared to the other causes, the risk of nonunion was higher in fractures resulting from car accidents (p=0.032). A statistically significant difference in the incidence of non-union was observed between the severity of fracture (p=0.013). The probability of nonunion was approximately 18.7 times higher when displacement was observed on lateral radiographic images after the primary surgery than when no displacement was observed, and this difference was statistically significant (p=0.004). Similarly, the probability of nonunion was 11 times higher when displacement was observed on AP radiographic images after the primary surgery than when no displacement was observed, and this difference was also statistically significant (p=0.014). Nonunion was found to be unrelated to the surgical method (implant).

A logistic regression analysis was performed after correcting for age, traumatic history, Seinsheimer fracture classification, and displacement in the AP and lateral views. The results showed that only the displacement in the AP and lateral views was a significant variable. The probability of nonunion was approximately 15.4 times higher when displacement was observed in the AP view (95% confidence interval [CI]: 1.33–176.82) and 24.2 times higher when displacement was observed in the lateral view (95% CI: 1.76–335.67).

## DISCUSSION

An IM nail is a device with numerous biomechanical advantages, but malreduction occurs easily in subtrochanteric fractures owing to the short proximal segment and wide medullary canal, which makes it difficult to achieve stable fixation. These features may be associated with nonunion.^12^,^14^ In the present study, 6 of the 7 nonunion cases showed displacement in the initial post operative radiographs.

When IM nails are inserted, because it is impossible to examine images in two planes at once with the C-arm, displacement can easily occur in at least one plane. In the authors’ experience, most techniques involve inserting the IM nail while looking at the AP image. Hence, displacement frequently occurs in the sagittal plane, equivalent to the lateral image. If the IM nail portal is not positioned correctly, the risk of displacement increases even further. If the start point is too anterior, an anterior apex angulation forms and the distal fragment is displaced posteriorly.^15^ In this case, not only do the medial and lateral sides of the subtrochanteric area receive mechanical stress but also the anterior and posterior cortical bones are subjected to abnormal stress.^12^ Hence, the fixation ability of an IM nail is difficult to consider as sufficient by itself in the wide medullary canal. Mechanical stress can cause excessive movement of the fracture, and this is thought to be a factor of union impairment.

In addition, the internal diameter of the subtrochanteric medullary canal is often longer in the AP direction than in the transverse direction. Thus, owing to the strong action of the psoas muscle, fixation with an IM nail is more likely to result in displacement in the sagittal plane than in the coronal plane.^16^,^17^ This is thought to have acted as a cause of nonunion, as in the present study.

**Fig.1:**
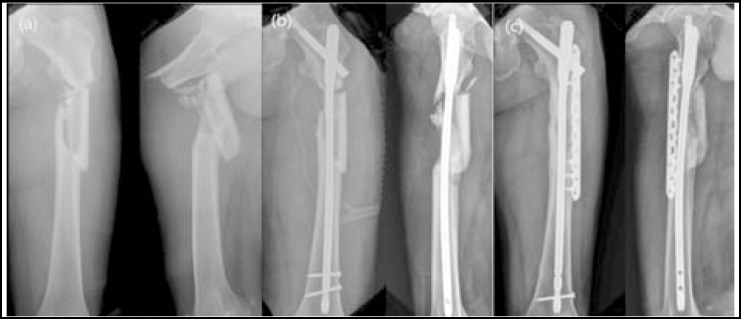
Post-injury image of ‘nonunion case 4’ showing that the fracture is comminuted and accompanied by flexion & external rotation of the proximal fragment (a). Image obtained immediately after primary surgery, showing displacement of the fracture in the AP and lateral views (b). Union is achieved after autogeneous iliac bone graft and additional plating (c).

Proper reduction is difficult for severely comminuted fractures, and so additional techniques are required, such as limited open reduction with clamp or cables.^8^,^18^ Ali et al.^19^ claimed that one implant was insufficient for fixation of severely comminuted subtrochanteric femoral fractures of Seinsheimer classification type IV or V, and so additional fixation was required. In the present study, all 7 cases that showed nonunion involved comminution, and five cases were Seinsheimer classification type IV or V. In particular, additional fixation is thought to be required for type IV fractures with a comminuted segmental fragment (Fig.1).

### Limitations of the study

The cases included in each category were too few to ensure sufficient statistical power. As this was a retrospective study and not all patients were treated by the same surgeon, implants and surgical methods could not be standardised. In addition, the patients’ functional results could not be included in the analysis.

Nevertheless, the fact that a statistically significant result was obtained even with a somewhat small number of cases is meaningful because it not only provides a reason to take interest in displacement of fractures but also suggests the need for more precise reduction during surgery. Furthermore, this is evidence that additional fixation is required when displacement cannot be overcome by intramedullary fixation.

When displacement is present after performing internal fixation for comminuted subtrochanteric fractures, the probability that stable fixation will not be achieved appears to be higher, meaning that the risk of non-union will increase.
